# Is perfectionism associated to dental anxiety?

**DOI:** 10.1192/j.eurpsy.2021.491

**Published:** 2021-08-13

**Authors:** A.T. Pereira, C. Cabaços, A. Araujo, M.J. Soares, A. Macedo

**Affiliations:** 1 Institute Of Psychological Medicine, Faculty Of Medicine, University of Coimbra, coimbra, Portugal; 2 Psychiatry Department, Centro Hospitalar e Universitário de Coimbra, Coimbra, Portugal

**Keywords:** Dental Anxiety, Perfectionism

## Abstract

**Introduction:**

Personality traits like neuroticism and trait-anxiety, as well as the predisposition to a greater sensitivity to pain, are risk factors for dental anxiety. Although perfectionism has been associated with both anxiety and pain, particularly when mediated by repetitive negative thinking/RNT (Macedo et al. 2015; Albuquerque et al. 2013), its role in dental anxiety has not yet been studied.

**Objectives:**

To analyze the role of perfectionism and RNT in dental anxiety.

**Methods:**

A community sample of 552 adults (68.2% women; mean age=35.15±15.79 years) completed the Portuguese versions of:Hewit and Flett Multidimensional Perfectionism Scale–13, State-Trait Anxiety Inventory, Sensitivity to Pain Traumatization/SPT Scale, Fear of Dental Pain/FDP Questionnaire, Perseverative Thinking Questionnaire and Dental Fear Survey/DFS.

**Results:**

Trait-anxiety (r=.225), socially prescribed perfectionism/SPP (r=.177), SPT (r=.286), FDP (r=.509) and RNT (r=.274) were significantly (p<.01) correlated with dental anxiety (DFS total score). Serial mediation analysis using the PROCESS-macro 3.5 for SPSS (Hayes, 2020; Model 6) showed that even controlling for trait-anxiety and gender (as SPT, FDP and RNT mean scores were significantly higher in women, p<.01) SPP plays a significant indirect effect through SDT, FDP and RNT on dental anxiety, which are (isolated or sequentially) full mediators of this relationship (Total effect: .553, p<.001).

**Conclusions:**

This study shows for the first time that negative perfectionism is a predictor of dental anxiety; its influence operates through the increase in levels of sensitivity to pain, DPA and RNT. We suggest that when intervening in this health problem it is important to evaluate perfectionism and try to mitigate its negative impact, namely diminishing RNT and the focus on pain and fear.

